# A cross-sectional study of COVID-19 testing and disclosure hesitancy: The role of responsibility attribution in South Korea

**DOI:** 10.1371/journal.pone.0330737

**Published:** 2025-08-21

**Authors:** Minjung Lee

**Affiliations:** 1 School of Nursing, Yale University, West Haven, Connecticut, United States; 2 Dental Research Institute, School of Dentistry, Seoul National University, Seoul, Korea; Chulalongkorn University Faculty of Medicine, THAILAND

## Abstract

Efficient and effective public health surveillance during epidemics relies heavily on active and voluntary public participation, including timely COVID-19 testing and disclosure of results to contacts. This study aimed to investigate predictors of COVID-19 testing and disclosure hesitancy, with a focus on the role of responsibility attribution during the early stages of the Omicron epidemic in South Korea. A cross-sectional survey was conducted with 1,000 participants between February 25 and March 2, 2022. Chi-square tests and multivariable logistic regression models were used for analysis. Findings showed that 41.5% of participants expressed hesitancy toward COVID-19 testing, and 59.4% expressed hesitancy toward disclosing test results to contacts. Greater attribution of responsibility to individuals was significantly associated with increased hesitancy toward testing (OR = 0.75, 95% CI = 0.63–0.90, p < 0.001) and disclosure (OR = 0.67, 95% CI = 0.56–0.80, p < 0.001). Conversely, testing acceptance was positively associated with trust in the government (OR = 1.29, 95% CI = 1.07–1.55, p = 0.01), social support (OR = 2.18, 95% CI = 1.73–2.73, p < 0.001), and full vaccination status (OR = 1.98, 95% CI = 1.11–3.50, p = 0.02). Disclosure acceptance was similarly associated with trust in the government (OR = 1.26, 95% CI = 1.05–1.51, p = 0.01) and social support (OR = 2.23, 95% CI = 1.77–2.81, p < 0.001). These results underscore the central role of responsibility attribution in shaping public participation in surveillance efforts. Mitigating excessive individual attribution and fostering a balanced perspective that integrates both personal and contextual factors may enhance public engagement. While attribution remains a key psychological predictor, building trust in government and strengthening social support systems also emerge as important strategies for promoting active and voluntary participation in public health surveillance.

## Introduction

South Korea’s public health surveillance strategy in response to the coronavirus pandemic during its early stages has been widely regarded as one of the most effective models worldwide. A key component of South Korea’s approach was the robust surveillance system known as the testing, tracing, and treating (3Ts) strategy against the coronavirus [1]. At the beginning of the epidemic, the Korea Disease Control and Prevention Agency (KDCA) established this strategy, which involved operating aggressive COVID-19 screening stations to identify patients at the earliest point possible and detect suspected cases, including those showing symptoms of COVID-19. The prompt tracing strategy aimed to prevent secondary and tertiary infections across the country by immediately identifying and notifying individuals who had been in contact with confirmed patients to seek testing [2,3]. As a result, South Korea managed to flatten the COVID-19 curve without resorting “lockdown” measures during the early stages of the pandemic.

However, when the Omicron variant became the dominant strain in February 2022, it caused significant local community transmission in South Korea, leading to an exponential increase in the number of cases. The average daily confirmed cases rose from approximately 6,000 in December 2021 (when Delta was dominant) to about 300,000 in March 2022 (when Omicron was dominant) [4]. In this situation, high intensity contact tracing became inefficient and ineffective. Consequently, South Korea’s surveillance policy shifted its focus from early detection to targeting high-risk groups. Public health officers no longer administered COVID-19 testing for symptomatic patients and ceased contact tracing; testing for close contacts of confirmed patients was no longer mandatory. Instead of contact tracing, patients were required to disclose their COVID-19 positivity to notify close contacts of exposure and potential infection.

This shift in public health surveillance necessitated active and voluntary engagement from the public, termed “participatory disease surveillance.” Participatory disease surveillance is described as “active” because it relies on individuals willingly and knowingly providing information crucial for public health action [5,6]. Public participation is integral to public health surveillance, requiring active involvement from individuals suspected of infection to provide epidemiological data and notify close contacts of their confirmation. Therefore, at that time, voluntary COVID-19 testing, and the disclosure of results became critical for public health surveillance.

However, when participation in surveillance is voluntary rather than mandatory, several barriers may arise. A rapid scoping review has shown that low health literacy, low trust in the healthcare system, the cost of testing, and the stigma and consequences of testing positive are significant barriers to COVID-19 testing [7]. In South Korea, testing positive led to seven days of isolation, resulting in restricted freedom, economic repercussions, and a reduced quality of life, which could act as barriers to testing. Moreover, disclosing a COVID-19 diagnosis can expose individuals to blame and significantly alter interpersonal relationships and the support they receive from their social network [8,9]. Consequently, it is understandable that individuals experiencing COVID-19 symptoms or having close contact with infected individuals may be hesitant to disclose their contact history and refuse testing. However, delayed disease detection can lead to disease progression to more severe stages and further spread during epidemics, posing challenges for disease control efforts [10].

This study seeks to identify the predictors that may enhance voluntary engagement in public health surveillance, such as testing and disclosure of infection status. By understanding predictors of participation, such as attribution of responsibility, trust in government, and social support, this study offers insights that can inform communication strategies and public health interventions aimed at improving positive surveillance outcomes. These findings can support the development of evidence-based messaging and policy approaches that encourage public cooperation in future outbreak scenarios.

### Predictors of COVID-19 testing and disclosure

Previous studies have explored the predictors of COVID-19 testing and found that such behavior is related to individuals’ characteristics. Males [11,12] and older individuals [11] show higher testing acceptance, while ethnic minorities [11,13] and those with lower socioeconomic status [12,14] exhibit more hesitancy in testing. Job status also influences COVID-19 testing, with unemployed/retired individuals [12], full-time workers [15], and those able to work if test results are positive [16] being more likely to get tested. Additionally, health-related factors such as comorbidities [11,12] are associated with higher testing intentions. Psychological factors, including perceptions of infection risk [17], knowledge of asymptomatic COVID-19 cases [15], and a desire to avoid infecting others [18], also correlate with higher testing intention. One early study during the COVID-19 pandemic (April 2020) assessed COVID-19 stigma and testing intentions, identifying a significant association between anticipated stigma and COVID-19 testing intentions [19].

Limited studies have investigated predictors of disclosure of confirmed COVID-19 patients, and most of these studies are qualitative. Non-disclosure issues were commonly reported during the COVID-19 outbreak. The reasons for this may be similar to other infectious diseases that have reported which reported stigma against the patients (e.g., leprosy, influenza, or severe acute respiratory syndrome) [20–22]. According to a qualitative study that explored disclosure experience among confirmed patients in China, the main reasons for disclosure were government policy; social responsibility; gaining support; and fear of being blamed for nondisclosure [23]. A phenomenological study conducted in Malaysia revealed experiences among confirmed patients after disclosure of being isolated, labeled, and blamed by the people surrounding them, including healthcare providers, neighbors, and service-counter staff [20].

### Attribution of responsibility on infection control

Weiner’s (1985) attribution model assumes that people engage in causal searches after events in order to understand their occurrence. During causality quests, people assess different aspects of the perceived cause of an event, forming the basis for subsequent judgments and inferences of individual responsibility and blame [24,25]. This theory states that attribution of responsibility primarily depends on perceived controllability (i.e., whether an event is responsible), locus of causality (i.e., whether an event is caused by something internal or external), and stability (i.e., whether the event is enduring) [26]. These causal explorations guide emotional and behavioral responses to that event [27].

Individuals’ attribution of responsibility can impact public responses to events both positively and negatively. When the public perceives disease contraction as controllable by individuals, they are more likely to hold the infected individuals responsible for their illness, influencing how people cope with the event [28]. This attribution can encourage problem-solving activities at the individual level. For instance, a study from Italy suggested that causal beliefs influence the extent to which people adopt scientific preventive behaviors [29]. However, attributing responsibility can also lead to blame, stigma and social rejection of the infected individuals [30,31]. It affects affective responses, such as blaming those who fall ill or infect others with COVID-19 [26]. Attributing disease control to individuals increased personal responsibility and blame, leading to greater stigma [32]. The desire to punish someone who boarded a flight despite a positive COVID-19 test was higher when greater responsibility was attributed to that individual [33]. Additionally, attributing responsibility for infection to individuals can result in poor mental health [34]. However, the role of responsibility attribution on individuals’ mitigation behavior, especially COVID-19 testing behavior and disclosure of the results is still limited.

### Current study

In this current study, we investigate predictors of COVID-19 testing and disclosure hesitancy, focusing on the role of responsibility attribution during the early stages of the Omicron virus epidemic in South Korea (February 2022). Given the significant impact of sociodemographic characteristics on testing hesitancy reported in previous studies, we include these factors in our analytical model. Additionally, we examine health-related factors, including vaccination status. Our study incorporates two constructs from the Health Belief Model (HBM): perceived susceptibility to COVID-19 and perceived severity of infection, known determinants of health behaviors [35–37]. Trust in governmental control measures is also assessed as a potential contextual factor influencing COVID-19 testing and information disclosure. Previous literature has shown associations between trust in governmental responses to COVID-19 and the adoption of preventive measures [38,39]. Specifically, our study aims to (1) assess the level of COVID-19 testing and disclosure of positive results, (2) identify predictors of these behaviors, and (3) examine the impact of responsibility attribution on these behaviors. By identifying factors that influence participation in testing and disclosure, this study contributes to understanding how to promote voluntary engagement in public health surveillance.

## Methods

### Study design

We conducted an online cross-sectional survey from February 25-March 2, 2022, about two years after the Korea Centers for Disease Control and Prevention (KCDC) confirmed the first case at the early stage of the epidemic. This study was performed when the Omicron variant detection rate among confirmed South Korean cases of COVID-19 was more than 90%. An anonymous online questionnaire was developed to evaluate the public’s intention to participate in testing and disclosure of positive results and investigates participants’ sociodemographic and health-related factors, as well as psychological factors, including attribution of responsibility, perceived risk, and trust. The original English questionnaire was translated into Korean by a bilingual research team, with cultural and linguistic adaptations to ensure conceptual equivalence and clarity within the South Korean public health context. The survey was conducted via an online platform from a survey research company. The company recruited respondents by sending survey invitations containing general information about the survey, including its purpose and an online consent statement via e-mail or text message, to registered nationwide survey panel members who met the inclusion criteria. Only participants who confirmed their consent after reviewing the statement were permitted to proceed with the questionnaire. The criteria required that each participant be: (1) aged 18 years or older, (2) a resident in South Korea, and (3) a Korean speaker. The company enrolled respondents using age, sex, and geographic region-based proportional and quota sampling process. 1,023 subjects completed the survey, and 1,000 were included in the analysis. Findings will be shared with relevant Korean public health stakeholders through academic presentations, collaborations, and public communications in Korean to facilitate accessibility of results.

### Measures

#### Dependent variables.

A 5-point scale was used to measure respondents’ intentions to get a COVID-19 test and disclose the results. Participants were asked how much they agreed with the following statements: “If I develop symptoms of suspected COVID-19 infection, I will have myself tested for COVID-19,” and “If I am confirmed with COVID-19, I will actively inform close contacts.” Responses were rated on a 5-point Likert-type scale, with “1 = Definitely not and 5 = Definitely.” For analysis, testing and disclosure variables were coded such that 1 indicated acceptance and 0 indicated hesitancy, although results are discussed in relation to hesitancy for consistency with the study’s focus.

#### Independent variables.

Sociodemographic factors included gender (0 = male, 1 = female), age, family size (i.e., living alone or with more than 2 persons), the presence of children (none = 0, more than one = 1), marital status (i.e., married, single, divorced/bereaved), and job status (self-employed, employed, other). We also assessed education level (1 = high school graduate or below, 2 = bachelor’s degree, 3 = graduate or professional degree) and monthly household income in South Korean won (1000 won = US $0.87; 1= < 2 million won, and 4= ≥ 6 million won).

Health-related factors included COVID-19 vaccination history, presence of underlying disease, subjective health, and previous COVID-19 diagnosis for the participants. For COVID-19 vaccination history, participants were asked, “How many times have you been vaccinated against COVID-19?” Responses included “None,” “Once,” “Twice,” and “Twice and also a booster shot.” We grouped the participants who completed their basic injection (“Twice” and “booster shot”) or not. Subjective health status (poor = 1, moderate = 2, good = 3) was investigated to assess health-related factors. We also investigated the presence of underlying disease by asking participants to indicate all diagnosed underlying diseases (e.g., hypertension, diabetes, chronic cardiac disease, asthma, cancer, etc.). We grouped the participants as being with or without diagnoses for one or more underlying diseases. For mental health, we asked whether the participant had visited a psychiatrist or received psychological counseling in the past year (yes = 1, no = 0).

Responsibility attribution of the disease were measured with three items which were adopted from Mak et al. [32]: controllability (“COVID-19 patients have the ability to control their infection of the disease”), responsibility (“COVID-19 patients are responsible for their own infection”), and blame (“It is the COVID-19 patients’ own fault that they have the disease”). Participants indicated whether they agreed with the above items on a 5-point Likert scale, with higher scores indicating greater personal responsibility to the disease, and more blame directed to the infected individuals. For analysis, we used the average value of the three aspects.

To measure attitudes related to COVID-19, we examined the perceived risk of COVID-19 infection (two items) comprising perceived susceptibility, which signifies individuals’ beliefs about the possibility of infection and the perceived severity of the infection [35]. Respondents answered, “What do you think is the likelihood of your COVID-19 infection?” and “What do you think will be the severity if COVID-19 infects you?” Responses were rated on a 5-point Likert-type scale, with “1 = very low, 3 = neither low nor high, and 5 = very high” (Table 4). We also investigated participants’ trust in the government by asking, “To what extent do you currently trust the government to respond to infectious diseases?” Responses were collected using a 4-point scale, with “1=not at all to 4=very much.” We also investigated social support (4 items), asking, first, how many people they can ask for help when life tasks (e.g., livelihood, housework, childcare, etc.) increase due to COVID-19 and (2) when they need to make an important decision due to COVID-19, whether there is someone to turn to for support. Psychological measures were adapted from previously validated instruments used in pre-pandemic public health research. These measures were selected for their theoretical grounding and comparability, and internal consistency was confirmed using Cronbach’s alpha.

**Table 4:** Predictors of COVID-19 testing and disclosure acceptance.

	COVID-19 testing				Disclosure of positive results			
*Socio-demographics*	Exp(B)	95% C.I.		P	Exp(B)	95% C.I.		P
**Gender**	1.372	1.013	1.857	.041	1.541	1.139	2.084	.005
Male								
Female	.748	.443	1.261	.275	.893	.532	1.498	.667
**Age group**	1.136	.676	1.908	.630	1.373	.820	2.298	.228
20s	1.602	.875	2.931	.127	1.582	.864	2.898	.137
30s	1.641	.834	3.229	.152	1.119	.568	2.208	.745
40s								
50s	1.187	.839	1.681	.333	1.121	.793	1.585	.517
60s<	1.041	.611	1.773	.882	1.047	.616	1.778	.866
**Education level**								
High School graduate	1.223	.787	1.900	.371	1.166	.755	1.800	.489
College graduate	1.293	.788	2.120	.309	1.076	.660	1.755	.769
Graduate School	1.409	.859	2.310	.174	1.352	.828	2.205	.228
**Income level**								
200<	1.060	.684	1.644	.793	1.095	.708	1.692	.684
200-400								
400-600	1.001	.635	1.578	.995	.854	.543	1.343	.495
600>	1.228	.662	2.279	.515	.812	.436	1.512	.511
**Family size**								
More than two								
Living alone:1	1.035	.636	1.684	.891	.869	.532	1.419	.575
**Marital status**								
Single								
Married	1.785	1.189	2.679	.005	1.805	1.200	2.713	.005
Divorced/Bereaved	1.054	.669	1.662	.820	1.564	.995	2.460	.053
***Health-related factors***								
**Subjective health**								
Bad								
Moderate	.503	.328	.771	.002	.559	.366	.855	.007
Good	.739	.460	1.186	.210	.778	.485	1.249	.299
**Underlying disease**								
None								
More than 1	.932	.676	1.284	.666	1.345	.977	1.851	.069
**Mental disease**								
None								
More than 1	.739	.514	1.062	.102	.610	.423	.878	.008
**COVID-19 vaccinated**								
No								
Yes	1.976	1.115	3.502	.020	1.597	.926	2.756	.093
**Confirmed experience**								
No								
Yes	.904	.450	1.816	.776	1.147	.581	2.265	.692
***Psychological factors***								
Attribution of responsibility	.753	.632	.896	.001	.669	.562	.797	<0.001
Perceived susceptibility	.871	.725	1.047	.141	.974	.812	1.169	.781
Perceived severity	1.254	1.049	1.499	.013	1.145	.959	1.367	.134
Trust in government	1.289	1.073	1.547	.007	1.260	1.049	1.513	.013
Social support	2.176	1.732	2.735	<0.001	2.232	1.775	2.808	<0.001

### Ethical considerations

Approval of the Seoul National University Institutional Review Board was obtained before conducting the study (IRB No. 2202/004–017). Respondents provided electronic informed consent that appeared on the first page of the survey by answering a “Yes or No” question before being allowed to complete the online self-reporting questionnaire. The company that conducted the online survey protected the confidentiality of anonymous respondents.

### Statistical analysis

We conducted statistical analyses using R version 4.0.2 (R Foundation for Statistical Computing, Vienna, Austria). All results of quantitative variables were reported either as mean (M), standard deviation (SD), or frequency (percentage %) (Table 1). Differences in sociodemographic and health-related factors related to surveillance participation were examined using chi-square tests (Table 2). While continuous scores were used for psychological predictors, dichotomization was applied to the outcome variables to distinguish clear acceptance from hesitancy. This approach allowed the identification of participants with strong behavioral intentions (i.e., those who selected “definitely”), enhancing interpretability of the hesitancy frame used throughout the study. Internal consistency of multi-item psychological measures was confirmed using Cronbach’s alpha.

**Table 1:** Descriptive statistics of survey respondents (n = 1,000).

*Socio-demographics*	n	%
**Gender**		
Male	494	49.4
Female	506	50.6
**Age (years),** mean 47.22 (SD 15.15) years		
18-29	183	18.3
30-39	160	16
40-49	192	19.2
50-59	197	19.7
Over 60	268	26.8
**Family size**		
More than 2	837	83.7
1 (living alone)	163	16.3
**Marital status**		
Single	344	34.4
Married	570	57
Divorced/ Bereaved	86	8.6
**Presence of children**		
None	397	39.7
More than 1	603	60.3
**Education level**		
High school graduate or below	277	27.7
College	615	61.5
Graduate school and above	108	10.8
**Income level**		
<2	173	17.3
2-3.99	346	34.6
4-5.99	225	22.5
≥6	256	25.6
**Job status**		
Self-employed	170	17
Employed	522	52.2
Unpaid/Retired/Inoccupation	308	30.8
***Health-related factors***	n	%
**Subjective health**		
Bad	146	14.6
Moderate	512	51.2
Good	342	34.2
**Underlying disease**		
None	599	59.9
More than 1	401	40.1
**Mental disease**		
None	794	79.4
More than 1	206	20.6
**COVID-19 vaccinated**		
No	93	9.3
Yes	907	90.7
**Confirmed experience**		
No	957	95.7
Yes	43	4.3

**Table 2:** Responses of psychological factor variables.

Variable	M	SD	Range	Cronbach’s Alpha
Attribution of responsibility	2.73	0.85	1-5	0.79
Perceived susceptibility	3.16	0.88	1-5	–
Perceived severity	3.39	0.91	1-5	–
Trust in government	2.58	0.81	1-4	–
Social support	2.52	0.67	1-4	0.85

The psychological constructs measured in this study were drawn from previously validated instruments developed before the COVID-19 pandemic, enabling comparability with early-pandemic research and preserving theoretical continuity. The logistic regression models were used to analyze the associations between sociodemographic factors, health-related factors, and psychological factors including attribution responsibility and participants’ intentions to undergo testing and disclose positive results. Multicollinearity among independent variables was assessed using Variance Inflation Factor (VIF) values. A VIF threshold of 5 was used to indicate potential multicollinearity concerns.

## Results

### Sample characteristics

One-thousand individuals responded to the survey; the average age of participants was 47.22 years (SD = 15.15). Approximately half were females (50.6%), and 61.5% had a bachelor’s degree. Additionally, 34.6% of the respondents’ monthly household income ranged from 2 to 4 million KRW (34.6%), followed by over 6 million KRW (25.6%). Most were married (57.0%), had more than one child (60.3%), and had more than two household members (83.7%). About half of the respondents reported their job status as ‘employed (52.2%)’. Regarding health-related characteristics, about half perceived their health status as moderate (51.2%), while 14.6% responded as ‘bad.’ Among the respondents, 40.1% reported having more than one diagnosed health condition, and 20.6% reported visiting a psychiatrist or receiving psychological counseling in the past year (Table 1).

Responsibility attribution was measured using three items reflecting distinct dimensions of individual attribution: controllability, responsibility, and blame. Each item was rated on a 5-point Likert scale ranging from 1 (strongly disagree) to 5 (strongly agree). Among the three items, the mean score was highest for controllability (M = 2.83, SD = 0.97), followed by responsibility (M = 2.77, SD = 1.01), and blame (M = 2.57, SD = 1.06). A composite score representing overall attribution of responsibility was computed as the average of the three items (M = 2.73, SD = 0.85). The internal consistency of the scale was acceptable (Cronbach’s α = .79). Perceived susceptibility to COVID-19 was moderately high (M = 3.16, SD = 0.88), and perceived severity was slightly higher (M = 3.39, SD = 0.91), based on single-item measures using the same 5-point scale. Trust in government was measured using a single item on a 4-point scale, with a mean score of 2.91 (SD = 1.10). Social support was assessed using four items rated on a 4-point scale, capturing perceived emotional and instrumental support. The composite score demonstrated good internal consistency (Cronbach’s α = .85; M = 3.76, SD = 0.79) (Table 2).

### Prevalence of COVID-19 testing and disclosure hesitancy

To assess hesitancy, participants were asked whether they would get tested for COVID-19 if they developed symptoms, and whether they would inform close contacts if they tested positive. Responses were measured using a 5-point Likert scale: Definitely, Probably, Possibly, Probably not, and Definitely not. For analysis, only those who selected “Definitely” were categorized as non-hesitant (acceptance group). All other responses were classified as hesitant, reflecting uncertainty or unwillingness to engage in surveillance behaviors. Among respondents, 39.5% were classified as non-hesitant toward testing, having responded “Definitely,” while the remaining 60.5% fell into the hesitancy group. Regarding disclosure, 40.6% reported they would “Definitely” inform close contacts, while 59.4% expressed varying levels of hesitation.

Table 3 presents Chi-square statistics for group differences in testing and disclosure hesitancy across sociodemographic and health-related factors. For COVID-19 testing, individuals in their 30s (p = 0.02) and those in the lowest income group (< 2 million KRW; p = 0.03) were significantly more likely to be hesitant. By contrast, employed individuals showed the lowest hesitancy rates (p = 0.01). Among health-related factors, vaccination status was significantly associated with hesitancy: only 20.4% of unvaccinated participants reported definite testing intention, compared to 41.5% of vaccinated participants.

**Table 3:** Chi-square statistics for variables related to COVID-19 testing and disclosure.

		COVID-19 testing					Disclosure of positive results				
	n	Hesitancy		Acceptance		P	Hesitancy		Acceptance		P
**Gender**		n	%	n	%		n	%	n	%	
Male	494	305	61.7%	189	38.3%	0.43	312	63.2%	182	36.8%	0.02
Female	506	300	59.3%	206	40.7%		282	55.7%	224	44.3%	
**Age group**											
20s	183	117	63.9%	66	36.1%	0.02	114	62.3%	69	37.7%	0.05
30s	160	112	70.0%	48	30.0%		108	67.5%	52	32.5%	
40s	192	116	60.4%	76	39.6%		108	56.3%	84	43.8%	
50s	197	107	54.3%	90	45.7%		104	52.8%	93	47.2%	
60s<	268	153	57.1%	115	42.9%		160	59.7%	108	40.3%	
**Education level**											
High School graduate	277	173	62.5%	104	37.5%	0.70	169	61.0%	108	39.0%	0.81
College graduate	615	366	59.5%	249	40.5%		361	58.7%	254	41.3%	
Graduate School	108	66	61.1%	42	38.9%		64	59.3%	44	40.7%	
**Income level**											
200<	173	119	68.8%	54	31.2%	0.03	114	65.9%	59	34.1%	0.05
200-400	346	211	61.0%	135	39.0%		205	59.2%	141	40.8%	
400-600	225	136	60.4%	89	39.6%		139	61.8%	86	38.2%	
600>	256	139	54.3%	117	45.7%		136	53.1%	120	46.9%	
**Family size**											
More than two	837	503	60.1%	334	39.9%	0.55	495	59.1%	342	40.9%	0.70
Living alone:1	163	102	62.6%	61	37.4%		99	60.7%	64	39.3%	
**Marital status**											
Single	344	220	64.0%	124	36.0%	0.23	210	61.0%	134	39.0%	0.41
Married	570	332	58.2%	238	41.8%		329	57.7%	241	42.3%	
Divorced/Bereaved	86	53	61.6%	33	38.4%		55	64.0%	31	36.0%	
**Presence of children**											
None	397	226	56.9%	171	43.1%	0.06	226	56.9%	171	43.1%	0.20
Having children:1	603	379	62.9%	224	37.1%		368	61.0%	235	39.0%	
**Vaccination status**											
None	93	74	79.6%	19	20.4%	<0.001	70	75.3%	23	24.7%	<0.001
Vaccinated	907	531	58.5%	376	41.5%		524	57.8%	383	42.2%	
**Job status**											
Self-employed	170	114	67.1%	56	32.9%	0.01	117	68.8%	53	31.2%	0.02
Employed	522	291	55.7%	231	44.3%		295	56.5%	227	43.5%	
Other	308	200	64.9%	108	35.1%		182	59.1%	126	40.9%	

Regarding disclosure, significant group differences were observed by gender (p = 0.02), age (p = 0.04), income level (p = 0.04), and job status (p = 0.02). Similar to testing, those who had not been fully vaccinated reported the highest levels of disclosure hesitancy (with only 24.7% expressing definite intention to disclose). However, no significant differences were observed based on previous COVID-19 diagnosis for either testing or disclosure hesitancy (Table 3).

### Predictors of COVID-19 testing and disclosure hesitancy

We used logistic regression models to examine the association between testing and disclosure hesitancy and respondents’ sociodemographic, health-related, and psychological factors, including responsibility attribution (Table 4). Prior to running the models, multicollinearity was assessed using variance inflation factors (VIFs). All VIF values ranged from 1.07 to 1.62, indicating no multicollinearity concerns among the independent variables. Among sociodemographic factors, being female (OR = 1.37, 95% CI = 1.01–1.86, p = 0.04) and being employed (OR = 1.78, 95% CI = 1.19–2.68, p = 0.01) were associated with less hesitancy toward testing. Among health-related factors, participants who perceived their health as moderate were more hesitant to get tested (OR = 0.50, 95% CI = 0.33–0.77, p < 0.001), while those who were fully vaccinated were significantly less hesitant (OR = 1.98, 95% CI = 1.11–3.50, p = 0.02).

Higher attribution of responsibility to individuals was associated with greater hesitancy toward testing (OR = 0.75, 95% CI = 0.63–0.90, p < 0.001), suggesting that those who placed more blame on infected individuals were less likely to get tested themselves. In contrast, perceiving COVID-19 as more severe (OR = 1.25, 95% CI = 1.05–1.50, p = 0.01), greater trust in the government (OR = 1.29, 95% CI = 1.07–1.55, p = 0.01), and stronger social support (OR = 2.18, 95% CI = 1.73–2.73, p < 0.001) were all associated with reduced hesitancy toward testing.

Regarding disclosure of positive test results, being female (OR = 1.54, 95% CI = 1.14–2.08, p = 0.01) and being employed (OR = 1.80, 95% CI = 1.20–2.71, p < 0.001) were linked to lower hesitancy in informing close contacts. Participants who perceived their health as moderate (OR = 0.56, 95% CI = 0.37–0.86, p = 0.01) and those who had received mental health services during the previous 12 months (OR = 0.61, 95% CI = 0.42–0.88, p = 0.01) were more hesitant to disclose a positive result. Similarly, greater attribution of responsibility was associated with increased hesitancy to disclose (OR = 0.67, 95% CI = 0.56–0.80, p < 0.001), while greater trust in the government (OR = 1.26, 95% CI = 1.05–1.51, p = 0.01) and stronger social support (OR = 2.23, 95% CI = 1.77–2.81, p < 0.001) were associated with less hesitancy toward disclosure. Notably, having had a previous COVID-19 diagnosis was not significantly associated with hesitancy toward either testing or disclosure.

## Discussion

Our findings reveal that despite two years of exposure to the risk of coronavirus, a significant degree of hesitancy persists regarding COVID-19 testing and informing close contacts of confirmed cases. This study highlights that attributing responsibility to individuals acts as a barrier to participation in surveillance, COVID-19 testing, and disclosing positive test results. Conversely, trust in government, perceived risk levels, and social support facilitate public participation. Sociodemographic factors such as gender and job status, along with health-related factors including subjective health, mental health, and vaccination status, were found to influence both behaviors.

Several findings offer valuable insights. First, attributing infection responsibility to individuals inhibits rather than facilitates individuals from COVID-19 testing and disclosure of positive results. Studies examining the relationship between responsibility attribution and testing behaviors are scarce, but studies on blame allow us to understand what this result implies. When an epidemic spreads through a community, fear and stigma form cyclical patterns, people tend to blame the patients, leading to more negative consequences [40,41]. If the community tends to blame patients infected with COVID-19, people then begin to fear being blamed or stigmatized even more than their health condition [42]. Blaming reduces health behavior adaptation and infectious disease prevention [10,43], and also makes people defensive and worried [44]. These behaviors, in turn, delay detection, treatment, civic engagement, epidemiological investigations, and quarantine [21]. Individuals who assign higher levels of personal responsibility to COVID-19 patients were more likely to blame them [32], and this study’s findings support this notion. However, hesitancy to participate in testing and/or concealing relevant information due to fear of being blamed can lead to greater virus contagion [45].

Second, the results of this study also suggest factors that promote individuals’ testing and disclosure of positive results. The association of social support and trust in government with participation in public health efforts is robust. Numerous studies report that feeling supported by one’s social network is related to demonstrating prosocial behaviors toward others; therefore, perceived social support predicts prosocial behavior [46,47]. Specific features of social engagement, such as giving and receiving social support and prosocial behavior that benefits others, are considered because they can reduce the psychological harm associated with disasters, crises, and other stressful situations [48]. Research indicates that both giving and receiving social support are associated with decreased mental health symptoms [49,50], better stress coping strategies, which is particularly important during isolation and quarantine [51]. Thus, these forms of social engagement not only benefit health and well-being but also participation in surveillance; in turn, these behaviors may serve as protective factors during times of crisis [50].

In this study, trust in the government was also an important factor in promoting testing and disclosure of positive results. Numerous studies have shown that trust is highly relevant to perceptions of and willingness to participate or comply with institutional policies like vaccination, testing and contact tracing [52–54]. These observations are especially true where participation involves risks for individuals engaging in certain behaviors without guaranteeing reciprocity or appropriate behavior by others [55]. In other words, if the public trust an agency’s policies and believe that they are aligned with their interests, they are more likely to demonstrate trust and follow that agency’s advice [39,54].

Third, this study identified disparities and highlighted a subpopulation that exhibits higher levels of hesitancy towards COVID-19 testing and disclosure. Female participants, individuals in their 20s and 30s, and those in the lowest monthly household income group were less likely to participate in COVID-19 surveillance. Employed individuals showed higher rates of testing acceptance and information disclosure compared to self-employed and retired/unemployed individuals. These factors contribute to vulnerability within these sub-populations. The findings suggest that for many individuals and families, basic survival needs often take precedence in medical decision-making over prosocial motivations, such as preventing the spread of infection, which is consistent with findings from other studies [13,16].

Importantly, the findings of this study also highlight the vulnerability of unvaccinated people. Participants who have not finished their COVID-19 vaccines were about two times more likely to experience hesitancy toward COVID-19 testing than participants who finished their vaccination. According to an experimental study investigating the relationship between vaccination status and respondent attribution on confirmed patients, people attribute greater responsibility when unvaccinated people fall ill from or infect others with COVID-19 [26]. While unvaccinated people are at a greater risk of experiencing severe illness, hospitalization, and death from COVID-19 [56], our findings highlight that unvaccinated people can be even more vulnerable during the pandemic.

### Implications

The findings of this study have important implications for national public health protection and emergency preparedness planning. Conducted two years after the onset of the COVID-19 epidemic, the results confirm that high levels of hesitancy toward both COVID-19 testing, and disclosure remained. This emphasize the need for national strategies that foster voluntary public cooperation during health emergencies.

More specifically, the results highlight the importance of maintaining a balanced framing of responsibility attribution during national outbreaks. While emphasizing personal responsibility can promote precautionary behaviors, excessive internal attribution may discourage participation in public health surveillance. Media exposure can increase heuristic processing, which is fast, intuitive, emotional, and nonanalytical, shaping how people interpret messages about personal versus external responsibility [57,58]. Therefore, epidemic communication strategies should include careful attention to media narratives, alongside disease surveillance.

Finally, trust in national government institutions plays a critical role in sustaining public engagement. Participatory surveillance systems that rely on self-reported data through mobile or web-based tools require a strong foundation of public trust to function effectively. Since trust is more easily broken than built, particularly under conditions of uncertainty and risk [59], maintaining institutional trust must be central to any national epidemic preparedness framework.

### Limitation and future directions

This study has several limitations worthy of note. First, all measures were self-reported, which may introduce response bias, including social desirability and recall effects. Second, the study did not extensively explore the impact of stigma on public participation, but instead examined the role of responsibility attribution, which is known as a precursor to stigma. Third, as the study was cross sectional in design, it identified associations between attribution of responsibility and public participation in surveillance, rather than establishing causal inference. Fourth, some odds ratios were accompanied by relatively wide confidence intervals, which may reflect statistical variability or reduced precision in subgroup estimates and should be interpreted with caution.

Future research should explore the direct influence of stigma on surveillance participation, as well as potential mediation pathways between attribution, stigma, and public health behaviors. Additionally, exploratory analyses in this study tested interaction terms such as attribution by trust in government and attribution by social support, but no statistically significant interactions were found. These results were not included in the final manuscript to maintain clarity, but they point to an important area for further research on the combined effects of psychological and contextual predictors of surveillance behavior.

## Conclusion

The COVID-19 pandemic resulted in significant damage to the population, however, has provided many important lessons on public health surveillance for future epidemics. This study’s findings suggest that even after considerable period of exposure to the risk of coronavirus, a high degree of hesitation remains about COVID-19 testing and informing close contacts of confirmed cases. Responsibility attribution plays a role in public participation in surveillance, which reduces voluntary testing and disclosure of positive results, especially when active engagement of the public is required. Social disparities in public participation in surveillance were also identified, the vulnerable groups in need of priority assistance. Especially, unvaccinated people are less likely to get tested, which can lead to a greater risk of experiencing severe illness, hospitalization, and death from COVID-19. Building trust on government and social support can be the key to boost public participation in surveillance. Public health communication efforts including surveillance on media are recommended for an appropriate balance between external and internal attribution among public has been found.

## Supporting information

S1 File Inclusivity in global research questionnaire. (DOCX)
