# Supplementary material for: A cross-sectional study of COVID-19 testing and disclosure hesitancy: The role of responsibility attribution in South Korea
Source: PLoS One. 2025 Aug 21;20(8):e0330737. doi: 10.1371/journal.pone.0330737 (PMC12370103; doi:10.1371/journal.pone.0330737)
Supplement: S1 File — (DOCX) [file pone.0330737.s001.docx]

Inclusivity in global research

PLOS’ policy on inclusivity in global research aims to improve transparency in the reporting of research performed outside of researchers’ own country or community and ensures that PLOS publications reporting global research adhere to high standards for research ethics and authorship. Authors of relevant research articles may be asked to complete the questionnaire below, which outlines ethical, cultural, and scientific considerations specific to inclusivity in global research. This questionnaire may be requested when researchers have travelled to a different country to conduct research, if research uses samples collected in another country, research with Indigenous populations or their lands, or if research is on cultural artefacts. Researchers travelling to another country solely to use laboratory equipment will not normally be required to complete the questionnaire. However, the questionnaire can be requested at the journal’s discretion for any submission – if you have been requested to complete this questionnaire by the PLOS journal you submitted to, please do so.

Please complete the questionnaire below and include this as a Supporting Information file with your manuscript. Note that if your paper is accepted for publication, this checklist will be published with your article in the supporting information files. Please ensure that you reference the checklist in the main body of your manuscript. We suggest adding a subsection ‘Inclusivity in global research’ to your Methods section and adding the following sentence: “Additional information regarding the ethical, cultural, and scientific considerations specific to inclusivity in global research is included in the Supporting Information (SX Checklist)”

The questions have been designed to be applicable to a wide range of study types, and there are subsections for both human subjects research and non-human subjects research. If any of the questions are not relevant to your research please mark them as “N/A” as appropriate.

**Ethical considerations, permits and authorship**

*This section is applicable to all research types.*

Provide details as to who granted permissions and/or consent for the study to take place in the Methods section of your manuscript. This should include the names of **all** ethics boards, governmental organizations, community leaders or other bodies that provided approval for the study. If individuals provided approval refer to these people by their role or title but do not list their name(s).

Reported on page number: page 11

If there were any deviations from the study protocol after approval was obtained please provide details of these changes in the Methods section of your manuscript.
Did this study involve local collaborators that are residents of the country where the research was conducted or members of the community studied? If you do not have any authors from said communities, please provide an explanation for this below.

The study was conducted in accordance with the protocol approved by the Seoul National University Institutional Review Board (IRB No. 2202/004-017), with no deviations from the approved procedures.

This study did not involve local collaborators as co-authors. I am a Korean national and designed and conducted this research in South Korea while transitioning to my current position in the United States. The study received IRB approval in South Korea and was implemented with direct oversight and full awareness of the local cultural and public health context.

Everyone listed as an author should meet PLOS’ criteria for authorship and all individuals who meet these criteria should be included in the author byline, rather than the acknowledgements. For further information please see the journal’s Authorship Policy.

**Human subjects research (e.g. health research, medical research, cross-cultural psychology)**

Did you obtain written informed consent from a representative of the local community or region before the research took place? How did you establish who speaks for the community? Details of written informed consent obtained from study participants should be reported separately in the Methods section of your manuscript.

This study was conducted using an anonymous online survey with adult participants in South Korea. Given the nature of the study and the absence of direct interaction with identifiable community leaders or institutions, written informed consent from a community representative was not applicable. The study was reviewed and approved by the Institutional Review Board of Seoul National University (IRB No. 2202/004-017), which oversees ethical compliance for research involving human participants in South Korea. Informed consent was obtained electronically from all individual participants, as detailed in the Methods section of the manuscript.

How did members of the local community provide input on the aims of the research investigation, its methodology, and its anticipated outcome(s)?

As a Korean national with research experience in South Korea, I designed the study based on a deep understanding of the local public health context, cultural norms, and previous literature on COVID-19 risk perception and behavior. While members of the local community did not formally participate in shaping the study aims or methodology, the survey items were culturally adapted and reviewed by a bilingual research team familiar with Korean public health communication. The anticipated outcomes were guided by both theoretical frameworks and practical relevance to ongoing public health efforts in South Korea.

When engaging with the local community, how did you ensure that the informed consent documents and other materials could be understood by local stakeholders?

All study materials, including the informed consent statement and survey items, were written in Korean using language that was clear, culturally appropriate, and accessible to the general public. The materials were developed by a bilingual research team and reviewed to ensure conceptual and linguistic clarity. The informed consent document was included at the beginning of the online survey, and participants were required to confirm their understanding and agreement before proceeding.

Will the findings of the research be made available in an understandable format to stakeholders in the community where the study was conducted (e.g. via a presentation, summary report, copies of publications, etc.)? Please provide details of how this will be achieved.

I have published more than 20 peer-reviewed articles based on research conducted in South Korea prior to my transition to the United States, and I remain committed to sharing findings relevant to the Korean public health context. While the current study is being submitted to an international open-access journal, I plan to disseminate the results to Korean academic and professional audiences through future presentations, academic collaborations, and social media updates in Korean. The open-access publication format will also make the findings broadly available to public health stakeholders in South Korea.

**Non-human subjects research using specimens/ animals collected as part of the study, or those housed in archival collections. Examples include archaeology, paleontology, botany and zoology.**

Did the permission you obtained from a local authority to perform the study include an agreement on access to outputs and benefit sharing? This may include procedures to enable fair distribution of the benefits and resources arising from the research performed. Please include any details of Prior Informed Consent and Benefit Sharing Agreements obtained. These may be required by field-specific regulations, for example the Convention on Biological Diversity (CBD) and the associated Nagoya Protocol.

Not applicable. This study did not involve the collection of specimens or biological materials.

If the material used in your study was imported, please A) provide the year it was imported and B) indicate whether permits were obtained to import/export the materials used, C) provide details of any permits obtained. If this information is not available, please indicate this.

Not applicable. No imported materials or specimens were used in this study.

If you used archival specimens, please state how the material used in your study was acquired by the institute it is held in and provide details of any permits obtained for the original excavations/ sample collection. If this information is not available, please indicate this.

Not applicable. This study did not use archival specimens or physical materials.

How was the potential cultural significance of the materials collected in your study to local communities considered in your research design? Were Indigenous peoples and/or local researchers and institutions involved with archaeological excavations / collection of specimens? If so, please provide a description of their involvement.

Not applicable. The study was based on anonymous online survey responses and did not involve the collection of culturally sensitive materials.

If your manuscript includes photographs of human remains please indicate whether authors obtained permission from descendants or affiliated cultural communities to do so.

Not applicable. No human remains or images of human remains were used in this study.
